# Multi-walled carbon nanotubes induce COX-2 and iNOS expression via MAP Kinase-dependent and -independent mechanisms in mouse RAW264.7 macrophages

**DOI:** 10.1186/1743-8977-9-14

**Published:** 2012-05-09

**Authors:** Jong Kwon Lee, Brian C Sayers, Kyung-Soo Chun, Huei-Chen Lao, Jeanette K Shipley-Phillips, James C Bonner, Robert Langenbach

**Affiliations:** 1 Laboratory of Toxicology and Pharmacology, National Institute of Environmental Health Sciences, Research Triangle Park, Durham, NC, 27709, USA; 2 Toxicological Research Division, National Institute of Food and Drug Safety Evaluation, Korea Food and Drug Administration, Osong, 363-951, South Korea; 3 Department of Environmental and Molecular Toxicology, North Carolina State University, Raleigh, NC, 27695, USA; 4 College of Pharmacy, Keimyung University, Dae-Gu, 704-701, South Korea; 5 Department of Population Health and Pathobiology, Laboratory for Advanced Electron and Light Optical Methods, College of Veterinary Medicine, Raleigh, NC, 27695, USA; 6 Laboratory of Toxicology and Pharmacology, National Institute of Environmental Health Sciences, Research Triangle Park, P.O. Box 12233, Mail Drop C4-09, Durham, NC, 27709, USA

**Keywords:** Carbon nanotubes, Nanoparticles, Lung inflammation, Macrophages, Prostaglandins, Nitric oxide

## Abstract

**Background:**

Carbon nanotubes (CNTs) are engineered graphene cylinders with numerous applications in engineering, electronics and medicine. However, CNTs cause inflammation and fibrosis in the rodent lung, suggesting a potential human health risk. We hypothesized that multi-walled CNTs (MWCNTs) induce two key inflammatory enzymes in macrophages, cyclooxygenase-2 (COX-2) and inducible nitric oxide synthase (iNOS), through activation of extracellular signal-regulated kinases (ERK1,2).

**Methods:**

RAW264.7 macrophages were exposed to MWCNTs or carbon black nanoparticles (CBNPs) over a range of doses and time course. Uptake and subcellular localization of MWCNTs was visualized by transmission electron microscopy (TEM). Protein levels of COX-2, iNOS, and ERK1,2 (total ERK and phosphorylated ERK) were measured by Western blot analysis. Prostaglandin-E_2_ (PGE_2_) and nitric oxide (NO) levels in cell supernatants were measured by ELISA and Greiss assay, respectively.

**Results:**

MWCNTs, but not CBNPs, induced COX-2 and iNOS in a time- and dose-dependent manner. COX-2 and iNOS induction by MWCNTs correlated with increased PGE_2_ and NO production, respectively. MWCNTs caused ERK1,2 activation and inhibition of ERK1,2 (U0126) blocked MWCNT induction of COX-2 and PGE_2_ production, but did not reduce the induction of iNOS. Inhibition of iNOS (L-NAME) did not affect ERK1,2 activation, nor did L-NAME significantly decrease COX-2 induction by MWCNT. Nickel nanoparticles (NiNPs), which are present in MWCNTs as a residual catalyst, also induced COX-2 via ERK-1,2. However, a comparison of COX-2 induction by MWCNTs containing 4.5 and 1.8% Ni did not show a significant difference in ability to induce COX-2, indicating that characteristics of MWCNTs in addition to Ni content contribute to COX-2 induction.

**Conclusion:**

This study identifies COX-2 and subsequent PGE_2_ production, along with iNOS induction and NO production, as inflammatory mediators involved in the macrophage response to MWCNTs. Furthermore, our work demonstrates that COX-2 induction by MWCNTs in RAW264.7 macrophages is ERK1,2-dependent, while iNOS induction by MWCNTs is ERK1,2-independent. Our data also suggest contributory physicochemical factors other than residual Ni catalyst play a role in COX-2 induction to MWCNT.

## Background

Carbon nanotubes (CNTs) are engineered graphene cylinders that have numerous potential applications in engineering, electronics, medicine, and tissue engineering [1-4]. Single-walled carbon nanotubes (SWCNTs) are only a few nanometers in width whereas multi-walled carbon nanotubes (MWCNTs) consist of multiple cylinders concentrically stacked along a common long axis and can be 30 to 50 nm in width. Both SWCNTs and MWCNTs can be more than 10 micrometers in length, giving CNTs a high aspect ratio similar to many toxic fibers. Due to the increasing use of CNTs in a variety of products and applications, there is a concern that the emergence of these novel nanomaterials may cause new cases of occupational and environmental respiratory diseases [5-8]. Properties of CNTs that raise concerns of potential biological effects are their fiber-like shape which increases their persistence in tissues, the presence of residual metal catalysts from the manufacturing process (e.g., nickel, cobalt, and iron) that can generate reactive oxygen species (ROS), and high surface area per unit mass which further increases the potential for ROS generation [8-10]. CNTs have been shown to cause inflammation and fibrosis in the lungs of mice and rats [11-17]. However, the cellular and molecular mechanisms by which CNTs cause these diseases remain to be elucidated.

Two cyclooxygenases are known, COX-1 and COX-2; and both forms metabolize arachidonic acid into the family of lipid mediators called prostaglandins (PGs) [18]. COX-1 is constitutively expressed in tissues, whereas COX-2 is the inducible isoform. Both COX-1 and COX-2 have been shown to modulate lung inflammation [18], fibrosis [19], asthma [20], and carcinogenesis [21]. However, COX-2 is highly inducible by endogenous and exogenous stimuli and appears to be the major regulator of inflammation and pulmonary fibrosis [18,19]. The PG generated by COX-2 that mediates inflammation and plays a role in pulmonary fibrosis is thought to be PGE_2_. COX-2 is inducible by growth factors (e.g., PDGF, TGF-β1), cytokines (e.g., TNF-α, IL-1β) and oxidative stress. These stimuli also activate mitogen-activated protein kinase (MAPK) signaling. MAPK signaling has been reported to regulate COX-2 expression induced by radiation [22] or inflammatory stimuli such as bacterial lipopolysaccharide (LPS) [23]. In the latter report, LPS-induced COX-2 expression in the RAW264.7 macrophage cell line was reported to be partially inhibited by inhibitors of either ERK1,2 or p38 activation, but a combination of the two inhibitors was required to completely block LPS-induced COX-2 expression.

Inflammatory stimuli that activate COX-2 also activate inducible nitric oxide synthase (iNOS), which generates nitric oxide (NO). iNOS has also been reported to activate LPS-induced COX-2 in the RAW264.7 macrophage cell line by the endogenous generation of nitric oxide (NO) [24]. Futhermore, inhibitors of iNOS that reduce NO production also reduce PG production in cells [25]. Therefore, NO generated by iNOS activation during inflammation appears to be important in the activation of COX-2.

In this study we sought to determine whether carbon nanotubes, specifically MWCNTs, could induce COX-2 or iNOS expression in RAW264.7 macrophages through a MAPK-dependent mechanism and whether MWCNT-induced iNOS might influence the induction/activation of COX-2 by MWCNTs. We observed that MWCNTs caused ERK1,2 activation and COX-2 induction and that COX-2 induction was blocked by inhibition of ERK1,2. MWCNTs also induced iNOS and NO generation, however inhibition of iNOS did not significantly reduce MWCNT activation of ERK1,2 or induction of COX-2. Finally, we found that NiNPs induce COX-2 but that reducing the level of Ni in MWCNTs by 60% did not significantly reduce their ability to induce COX-2, suggesting that other factors in addition to Ni contribute to MWCNT induction of COX-2. These findings further elucidate the mechanisms through which novel engineered nanomaterials, such as MWCNTs, mediate an inflammatory response and should be useful for understanding the potential health risks they pose.

## Results

### Uptake and intracellular localization of MWCNTs in macrophages

**Figure 1:**
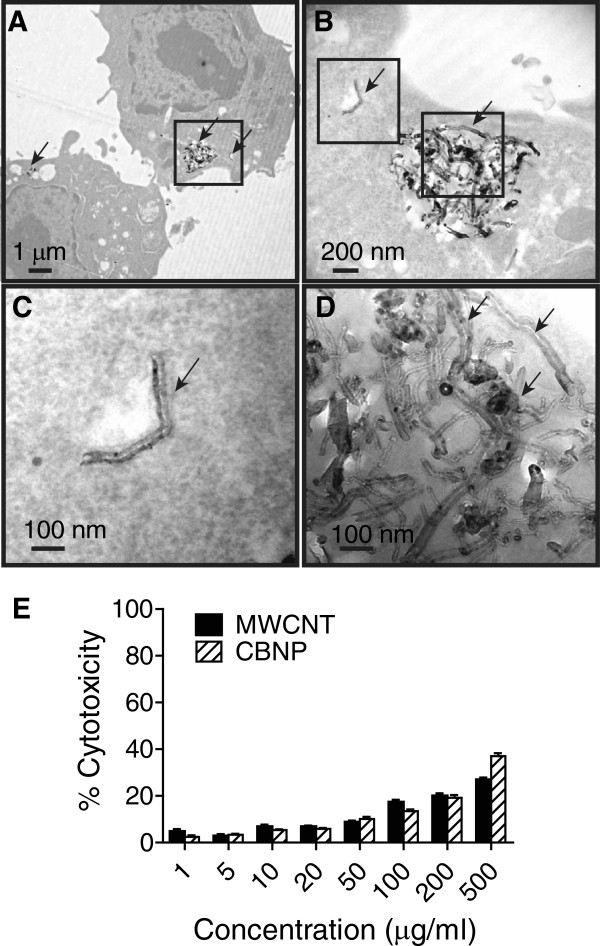
**TEM showing MWCNTs engulfed by RAW264.7 macrophages *in vitro.*** Cells were treated with MWCNTs (50 μg/ml) for 24 hr in serum-free defined medium and then fixed and embedded in agar and TEM performed as described in Methods. ***Panel A***: Low magnification showing two cells with MWCNTs within cytoplasm (arrows). ***Panel B***: Higher magnification of the inset frame from Panel A showing agglomerated MWCNTs and an individual MWCNT in cytoplasm (arrows). ***Panel C***: Magnification of left hand inset frame from Panel B showing individual MWCNT in cytoplasm. ***Panel D***: Magnification of right hand inset frame from Panel B showing aggregated MWCNTs (arrows). ***Panel E***: Cytotoxicity of MWCNT compared to CBNP in RAW264.7 cells as measured by LDH assay.

RAW264.7 macrophages engulfed MWCNTs in culture as shown by TEM (Figure 1A-D). MWCNTs were present within the cytoplasm of macrophages both as individual nanotubes (Figure 1B,C) and agglomerated nanotubes. (Figure 1B,D). The cytotoxic effects of MWCNTs and CBNPs on the RAW264.7 macrophages were determined using the LDH assay. Increasing concentrations of MWCNTs or CBNPs (1 μg/ml to 100 μg/ml) caused cytotoxic effects from 4% to 17%, respectively, at 24 hr as determined by the LDH assay (Figure 1E). Because the cytotoxicities of both MWCNTs and CBNPs were less than 20% at concentrations from 10 to 100 μg/ml, this dose range was used in subsequent experiments.

### MWCNTs induce COX-2 and iNOS and increase PGE_2_ and NO production

**Figure 2:**
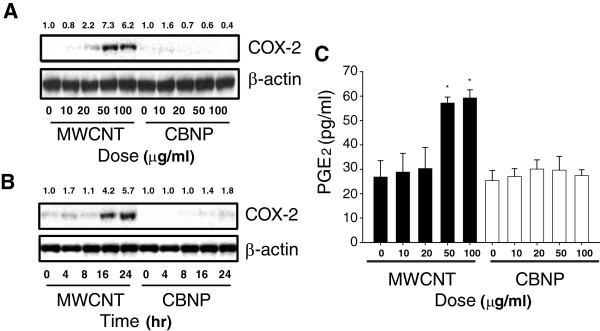
**MWCNTs, but not CBNPs, Induce COX-2 and PGE**_**2 **_**production by RAW264 macrophages *in vitro *in a dose- and time-dependent manner.** Western blot analysis showing **A)** dose response of MWCNT-induced COX-2 in RAW 264.7 cells. COX-1 was constitutively expressed by RAW264.7 and not changed by MWCNTs. **B)** Time course of COX-2 induction by 50 μg/ml MWCNTs in RAW264.7 cells. CBNPs used as a relatively inert control caused no induction of COX-2. Exposure of cells to MWCNTs or CBNPs and Western blotting was performed as described in Methods. **C)** PGE_2_ production after stimulation with MWCNTs or CBNPs. Cell supernatants were collected at 24 hr post-exposure and assayed by PGE_2_ ELISA as described in Methods. *Significantly different from the vehicle control (p < 0.05).The results shown are typical of two independent experiments. Numeric values above each Western blot represent the fold-increase in relative densitometric intensity of each COX-2 band relative to the control vehicle 1% pluronic surfactant (zero nanoparticle treatment) which was normalized to 1.0.

**Figure 3:**
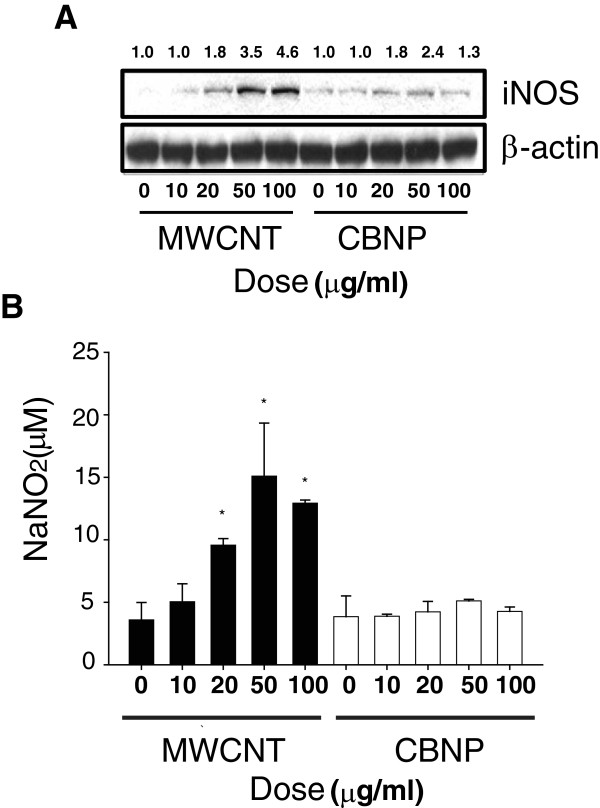
**Induction of iNOS and NO production by RAW264.7 cells exposed to MWCNTs**. Quiescent cultures of RAW264.7 cells were exposed to an increasing concentration of MWCNTs or CBNPs. **A)** Western blot analysis showed induction of iNOS protein with increasing concentrations of MWCNTs with no induction by CBNPs. Levels of β-actin are shown as a constitutive control protein in RAW264.7 cells. Numeric values above the Western blot represent the fold-increase in relative densitometric intensity of each iNOS band relative to control (zero) nanoparticle treatment which was normalized to 1.0. **B)** MWCNTs (solid bars), but not CBNPs (open bars), increased levels of sodium nitrite (NaNO_2_) in RAW264.7 cell supernatants as a measure of NO. *Significantly different from the vehicle control (p < 0.05).

MWCNT induction of COX-2 in RAW264.7 cells was dose-dependent and was maximal at 50 to 100 μg/ml MWCNT at 24 hr post treatment (Figure 2A). MWCNT (50 μg/ml) induction of COX-2 was also time-dependent and was maximal at 16 to 24 hrs post-treatment (Figure 2B). PGE_2_ levels in RAW264.7 cell supernatants were also significantly increased at 24 hr following treatment with 50 and 100 μg/ml MWCNTs (Figure 2C). In contrast, CBNP treatment at similar doses caused no increase in COX-2 or PGE_2_ levels. MWCNTs, but not CBNPs, also dose-dependently induced iNOS expression and nitric oxide (NO) production in RAW264.7 cells at 24 hr post treatment as measured by Western blot analysis and the generation of NaNO_2_ in cell culture medium, respectively (Figure 3A and B). Both iNOS induction and NO production were increased by MWCNTs at 20 μg/ml and maximally induced at 50 to 100 μg/ml.

### MWCNTs increase ERK1,2 activation and inhibition of ERK1,2 activation decreases COX-2 induction, but not iNOS induction

**Figure 4:**
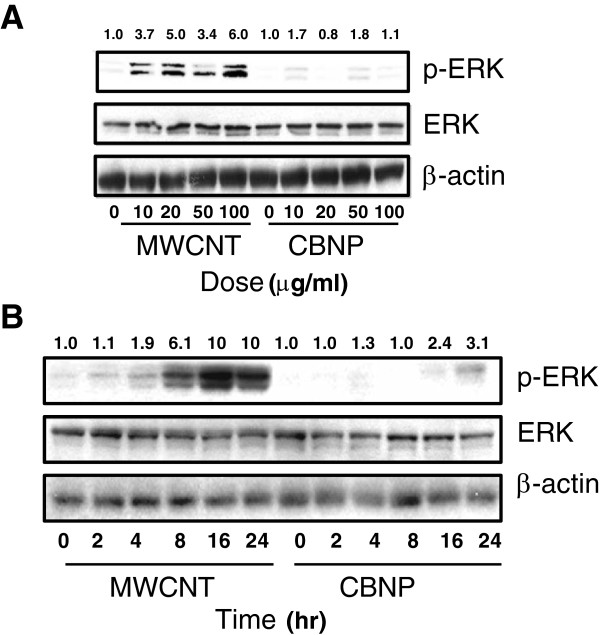
**Activation of ERK1,2 in RAW264.7 cells by MWCNTs. A)** Western blot analysis using an antibody specific for phosphorylated ERK (p-ERK1,2) showing that MWCNTs (50 μg/ml) increased ERK activation in a concentration dependent manner in RAW 264.7 cells. CBNPs did not cause a consistent concentration-dependent activation of ERK. Total levels of ERK1,2 protein and β-actin were not changed by MWCNTs or CBNPs. **B)** Western blot analysis showing time course of ERK phosphorylation by MWCNTs. Numeric values above each Western blot represent the fold-increase in relative densitometric intensity of each p-ERK1,2 band relative to control (zero) nanoparticle treatment which was normalized to 1.0.

**Figure 5:**
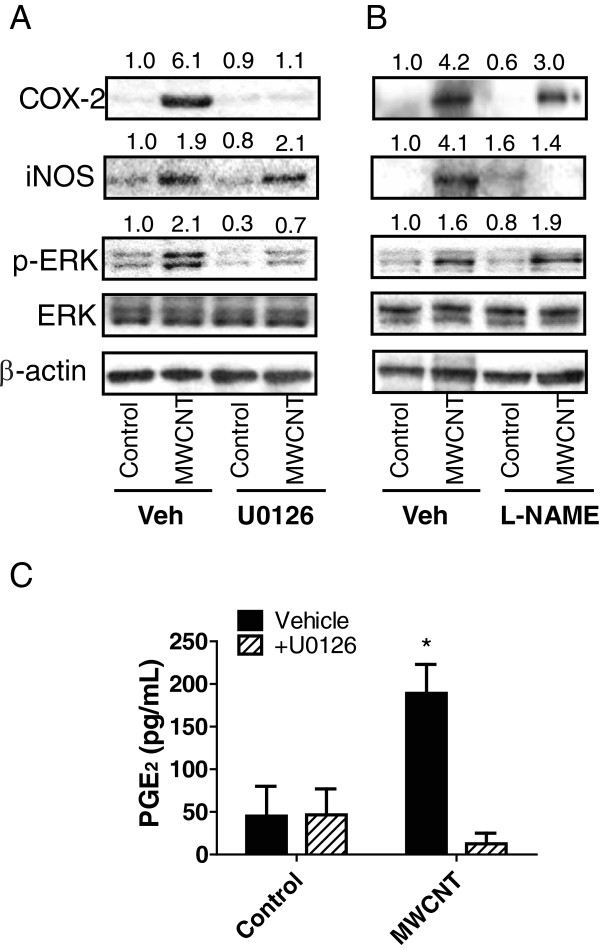
**Effect of pharmacologic inhibitors on MEK and iNOS on the induction of COX-2 in RAW264.7 cells exposed to MWCNTs. A)** Western blot analysis showing inhibition of COX-2 expression and phosphorylated ERK1,2 (p-ERK1,2) by the MEK inhibitor U0126 relative to vehicle (Veh) in cells treated with 50 μg/ml MWCNTs. Controls (C) were treated with serum-free medium alone. Levels of iNOS were not affected by U0126 (10 μM) at 24 hr post-treatment with MWCNTs, nor were constitutive levels of total ERK1,2 or β-actin. **B)** Western blot showing inhibition of MWCNT-induced iNOS at 24 hr by L-NAME (250 μM) with no significant effect on MWCNT-induced COX-2 or p-ERK. Numeric values above the Western blot represent the fold-increase in relative densitometric intensity of each band relative to control (zero) nanoparticle treatment which was normalized to 1.0. Data for all three experiments were from duplicate studies which yielded similar results. **C)** Results of PGE_2_ ELISA showing that production of PGE_2_ and release into cell supernatants after 24 hr treatment with MWCNTs (50 μg/ml) is blocked by 10 μM U0126 co-treatment.

MWCNTs, but not CBNPs, activated ERK1,2 phosphorylation in RAW264.7 cells in a concentration- and time-dependent manner. MWCNTs induced p-ERK1,2 formation at concentrations from 10 to 100 μg/ml when measured 24 hr after treatment (Figure 4A).A time course of activation showed that MWCNT (50 μg/ml) increased p-ERK1,2 from 4 to 8 hrs post-treatment, with maximal activation occurring 16 to 24 hr post-treatment. **(**Figure 4B). Inhibition of ERK1,2 activation by the MEK inhibitor U0126 inhibited COX-2 induction by MWCNTs, but did not affect the induction of iNOS (Figure 5A). As expected, U0126 (10 μM) blocked ERK1,2 phosphorylation induced by a 24 hr treatment with MWCNTs but did not change the level of ERK1,2 protein in RAW264.7 cells. The iNOS inhibitor L-NAME (250 μM) did not significantly reduce MWCNT-induced COX-2 induction 24 hr post-treatment nor did L-NAME reduce p-ERK (Figure 5B), but completely inhibited iNOS induction. The MEK inhibitor U0126 completely blocked MWCNT-induced PGE_2_ production by RAW264.7 cells at 24 hr post-treatment (Figure 5C).

### Role of residual nickel catalyst in mediating MWCNT induction of COX-2

**Figure 6:**
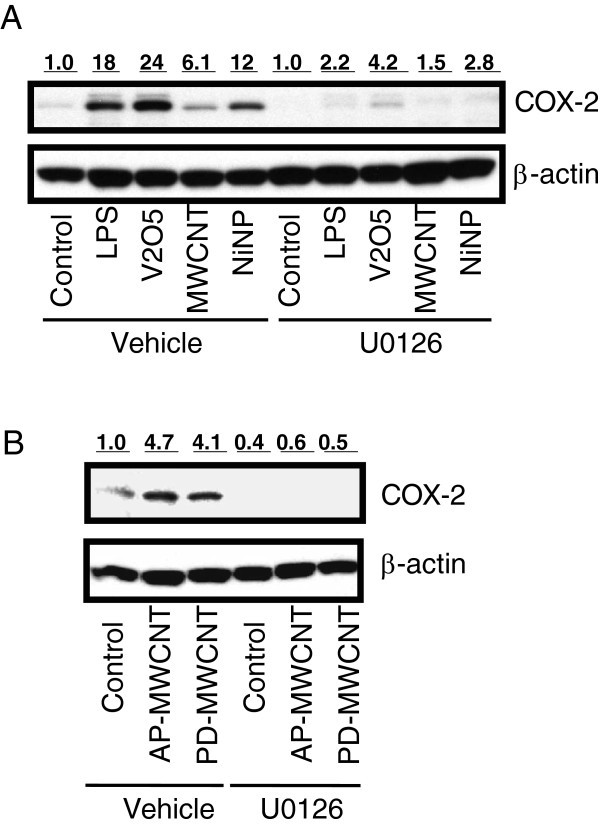
**COX-2 expression is induced by NiNPs in an ERK1,2-dependent manner in RAW264.7 cells, yet the Ni present in MWCNTs only partially mediates COX-2 induction. A)** Western blot showing COX-2 expression induced by NiNPs, MWCNTs, LPS, or V_2_O_5_ was significantly inhibited by U0126. Quiescent cultures of RAW264.7 cells were exposed to MWCNTs (50 μg/ml), NiNPs (10 μg/ml), LPS (0.1 μg/ml), or V_2_O_5_ (1 μg/ml) in the absence or presence of U0126 (20 μM) for 24 hr. **B)** Western blot showing induction of COX-2 by 50 μg/ml “as-prepared” (AP)-MWCNTs that contain 4.49% Ni and the same MWCNTs that have been purified (PD-MWCNTs) by acid washing to reduce Ni content to 1.8%. COX-2 induction was not significantly reduced by depletion of Ni catalyst. The MEK inhibitor U0126 blocked induction of COX-2 by either AP-MWCNTs or PD-MWCNTs. Numeric values above each Western blot represent the fold-increase in relative densitometric intensity of each COX-2 band relative to control (zero) nanoparticle treatment which was normalized to 1.0. Data are typical of at least two independent experiments.

The action of MWCNTs could be mediated, at least in part, by residual metal catalyst used in the manufacturing process. The MWCNTs used in this study contained residual NiNPs. As shown in Figure 6A, NiNPs increased COX-2 expression in RAW264.7 cells, and COX-2 induction by NiNPs, like MWCNTs, was significantly inhibited by the MEK inhibitor U0126. Similarly, the induction of COX-2 by LPS or V_2_O_5_ (two positive controls for COX-2 induction) was blocked by U0126. Further experimentation was conducted to determine the contribution of Ni in MWCNT in the induction of COX-2 in RAW264.7 cells. To accomplish this MWCNTs, pre- and post-acid washing were utilized. The pre-washed MWCNTs (AP-MWCNTs) contained 4.5% Ni, whereas the acid-washed MWCNTs (PD-MWCNT) contained 1.8% Ni, or approximately 60% less Ni. However, as shown in Figure 6B, COX-2 was similarly induced by MWCNTs containing both levels of Ni. Furthermore, inhibition of ERK1,2 activation effectively inhibited COX-2 induction by both AP-MWCNTs and PD-MWCNTs. Thus, a 60% reduction in Ni content did not affect the ability of MWCNTs to induce COX-2 or the involvement of ERK1,2 activation in their induction of COX-2.

**Figure 7:**
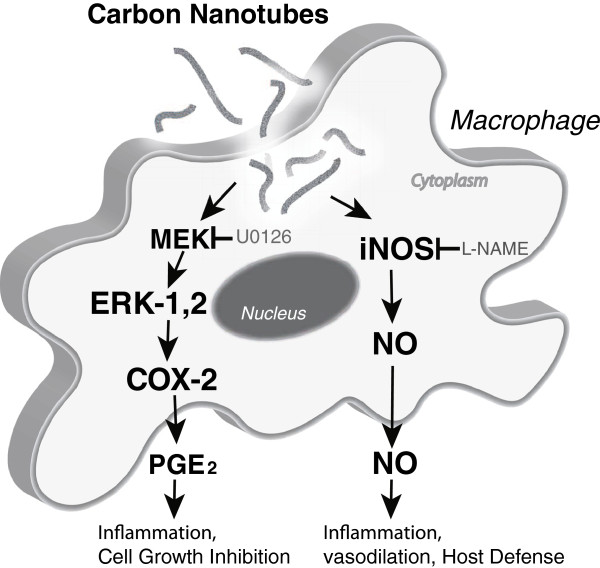
**Hypothetical scheme depicting uptake and intracellular signaling induced by MWCNTs leading to induction of COX-2, iNOS, and their respective secreted metabolites in RAW264.7 cells.** MWCNTs activate the MEK-ERK signaling cascade, which is required to induce COX-2 and production of PGE_2_. MWCNTs also induce iNOS and generation of NO through an ERK1,2-independent mechanism. The MEK inhibitor (U0126) and the iNOS inhibitor (L-NAME) are indicated along with their pharmacologic targets.

## Discussion

The increased expression and activity of COX-2 and iNOS in macrophages are two hallmarks of inflammatory and immune responses to a variety of stimuli, including LPS, metals, and oxidative stress. MWCNTs delivered to the lungs of mice by inhalation or oropharyngeal aspiration, or to rats by intratracheal instillation, are avidly engulfed by alveolar macrophages and MWCNT-containing macrophages are associated with progressive inflammatory and fibrotic lesions in the lung alveolar region, airways, or pleura of these animals [12,26,27]. In this study, we found that MWCNTs increased the expression of COX-2 and iNOS, and the induction of these two enzymes correlated with increased production of PGE_2_ and NO, respectively. Therefore, the induction of COX-2 and iNOS in RAW264.7 macrophages *in vitro* observed in the present study suggest that these enzymes and their products could play a role in the lung’s inflammatory or fibrogenic response to MWCNTs.

We further investigated upstream signaling that might mediate the induction of COX-2 and iNOS in RAW264.7 macrophages and found that MWCNTs increased the expression of COX-2 via an ERK1,2-dependent mechanism as demonstrated by blocking ERK activation with the MEK inhibitor U0126. While COX-2 expression was blocked by U0126, there was no discernable effect of U0126 on MWCNT-induced iNOS levels. MAPK signaling has been reported to regulate LPS-induced COX-2 expression in RAW264.7 cells [23]. However, LPS-induced COX-2 expression was partially blocked by inhibitors of ERK1,2 or p38 MAP kinase and combined blockade of these two kinases was required to completely inhibit COX-2 expression [23]. In the present study we demonstrated that COX-2 induction in RAW264.7 macrophages by LPS, V_2_O_5_, NiNPs, or MWCNTs was significantly inhibited by treatment with U0126, indicating that diverse organic and inorganic stimuli are able to induce COX-2 via ERK1,2-dependent signaling. In addition, we did not observe increased JNK or p38 MAP activation in RAW264.7 cells following MWCNT treatment (data not shown). Taken together, these findings suggest that ERK1,2 is the major pathway for MWCNT induction of COX-2 expression in these cells. However, a caveat of our data is that ERK was phosphorylated by relatively low concentrations of MWCNT compared to COX-2 induction (Figures 2 &4). These findings suggest that ERK phosphorylation is required but perhaps not sufficient to induce COX-2 at low MWCNT doses in RAW264.7 cells. Possibly at low MWCNT doses other intracellular signaling intermediates could play contributory roles in COX-2 induction. For example, NFκB and C/EBPbeta have been reported to mediate air pollution particulate matter-induced COX-2 expression in human bronchial epithelial cells [28].

The biological effects of MWCNTs could be due to multiple factors, including aspect (length to width) ratio, surface properties, aggregation or dispersion, and residual metal catalysts. For example, the purification of MWCNTs to remove residual metal catalysts used in the manufacturing process reduces the toxicity and pro-fibrogenic activity of MWCNTs [29]. Our results show that NiNPs are a potent inducer of COX-2. This suggests that at least part of the bioactivity of the MWCNTs used in our study could be due to residual Ni from the manufacturing process. While relatively high concentrations of Ni clearly induced COX-2 (Figure 6A), removal of ~60% of Ni from MWCNT (4.49% Ni in AP-MWCNT reduced to 1.8% Ni in PD-MWCNT) did not have a significant effect on MWCNTs ability to induce COX-2 induction by MWCNT (Figure 6B). Other groups have shown that the high aspect ratio (i.e., length) of MWCNTs, as well as other nanomaterials such as nickel nanowires, is perhaps the most important factor in determining macrophage activation, clearance, and ultimately disease outcome [9,30]. Given the data presented in Figure 6B we speculate that other factors in addition to Ni (e.g., nanotube length) are important to COX-2 expression in macrophages. However, as acid purification did not remove all residual nickel and even purified samples are not completely metal-free, Ni may still have a role in the induction of COX-2 in our studies. Furthermore, the metal catalysts present in MWCNT may not be bioavailable [31]. For example, the Ni present in MWCNTs appears to be encapsulated by carbon as observed by TEM (unpublished observation). Therefore, the relative contribution of Ni, nanotube length, and perhaps other factors, to COX-2 induction requires further study.

It is unknown whether ROS generation is involved in MWCNT induction of COX-2. MWCNTs have been reported to increase ROS production in lung cells *in vitro*[32,33]. It has also been shown that particulate matter-induced ROS generation is primarily of mitochondrial origin and results in increased COX-2 expression and IL-6 release by cultured bronchial epithelial cells [28]. In addition, the organic diesel exhaust constituent 1,2-napthoquinone caused mitochondrial production of H_2_O_2_ and increased levels of COX-2 and IL-8, both of which were diminished by the over-expression of catalase, which degrades H_2_O_2_[34]. We previously reported that vanadium pentoxide-induced H_2_O_2_ production in human lung fibroblasts occurs via NADPH oxidases [35]. Furthermore, p-ERK1,2, which was shown to mediate MWCNT-induced COX-2 in the present study, is also strongly activated by H_2_O_2_ in lung myofibroblasts [36]. While it is possible that MWCNTs induce ERK1,2-dependent COX-2 expression via ROS generation, the origin of ROS generation is complex and elucidation of ROS involvement in MWCNTs activity will require further study.

Others have reported that iNOS or reactive nitrogen species (RNS) generated by iNOS influence COX-2 activity. For example, iNOS activates COX-2 in LPS-stimulated RAW264.7 cells through generation of NO [24]. Furthermore, iNOS inhibitors have been reported to reduce PG production in carrageenan-induced inflammation in rats [25]. Based on these studies, cross-talk between iNOS and COX pathways has been proposed as an important contributing mechanism for inflammatory diseases [37]. However, in the present study the inhibition of iNOS with L-NAME did not significantly reduce MWCNT-induced COX-2 levels in RAW264.7 cells.

Both protective and pathogenic roles for COX-2 and its metabolites have been proposed. For example, PGE_2_ generated by COX-2 inhibits fibroblast and epithelial cell growth and reduces platelet-derived growth factor receptor expression in rat lung fibroblasts *in vitro*[38,39]. Furthermore, COX-2 knock-out mice display more inflammation and fibrosis in response to metals or allergens [19,20]. Our unpublished observations also show that COX-2 knock-out mice have exaggerated airway inflammation and production of IL-13 after combined exposure to ovalbumin and MWCNTs. Collectively these transgenic mouse models suggest that COX-2 is protective in lung inflammation and fibrogenesis. It is also noteworthy that patients with idiopathic pulmonary fibrosis have reduced levels of COX-2 [40]. Despite the evidence that COX-2 is protective in lung inflammatory and fibrotic diseases, there is also evidence that COX-2 and its metabolites have detrimental roles in mediating the pathogenesis of other diseases, particularly in arthritis and cancer [18,21]. Therefore, the significance of COX-2 in the pathogenesis of MWCNT-induced lung disease is unclear at present.

Both beneficial and potentially detrimental effects have also been ascribed to NO. It is well established that NO exerts a beneficial role through its action as a vasodilator and exogenous NO has been proposed to have therapeutic value for the treatment of asthma [41-43]. On the other hand, NO could have potential deleterious effect as it forms the highly toxic peroxynitrite (ONOO^-^) in the presence of H_2_O_2_ and acts as a potent signaling intermediate; causing tyrosine nitration and the activation of the EGF receptor and MAPK signaling pathways [36]. The generation of reactive nitrogen species derived from NO metabolism plays important roles in particulate-induced lung disease [44]. ONOO^-^ in particular has been implicated in the pathogenesis of lung and pleural disease associated with asbestos fibers [45,46]. Because MWCNTs have been compared to asbestos fibers with respect to their pathogenicity, it will be important to further elucidate whether MWCNTs are capable of generating RNS such as ONOO^-^. Our data show that MWCNTs increase NO and others have reported that MWCNTs increase ROS in lung cells [32,33]. Therefore, if NO and ROS are generated simultaneously then it is likely that ONOO^-^ will be formed in the lungs of rodents or humans exposed to MWCNTs.

Whether or not COX-2 and its metabolites, or iNOS-generated NO, are beneficial or detrimental following MWCNTs exposure remains to be elucidated. COX-2 deletion in mice results in susceptibility to metal-induced lung fibrosis [19] or allergen-induced lung inflammation [20], and the severity of lung inflammation in COX-2-deficient mice is due to reduced PGE_2_ production [47]. MWCNTs also induce fibrogenesis in the lungs of exposed mice and rats [7] and impair lung function [48]. Therefore, it would be important to determine whether MWCNT-induced inflammation and fibrosis are altered in the lungs of COX-2 deficient mice.

Modifications to alter the surface properties of MWCNTs could also alter biological activity. It has been recently shown that modification of MWCNTs by addition of carboxyl groups, as well as the dispersal state of MWCNTs affect the fibrogenic cellular responses that correlate with the extent of fibrosis in mice [29]. Furthermore, modifications of other types of nanoparticles, such as silica, by the addition of carboxyl or amine groups, changes the surface properties of these nanoparticles to alter intracellular localization and cytotoxicity in macrophages [49]. Therefore, COX-2 or iNOS induction in cultured cells could be indicative of the inflammatory or fibrogenic activity of an increasing diversity of CNTs or other engineered nanoparticles. Such information should allow us to better predict relative toxicity to humans and this information should aid in the design of safer nanomaterials.

## Conclusion

In the present study we showed that MWCNTs induce COX-2 expression and subsequent PGE_2_ production in RAW264.7 macrophages through a ERK1,2-dependent mechanism. MWCNTs also induced iNOS and NO production through an ERK1,2-independent process. Inhibition of iNOS partially blocked MWCNT-induced COX-2 expression, suggesting that NO generated from iNOS could serve to increase or stabilize COX-2 levels in macrophages. These findings further elucidate the molecular mechanisms involved in the macrophage response to MWCNTs and should be useful for understanding the molecular targets of carbon nanotubes and the potential health risks they pose.

## Methods

### Carbon nanotubes, carbon black, and nickel nanoparticles

For all experiments except where noted, the source of MWCNTs was Helix Material Solutions, Inc. (Richardson, TX). These MWCNTs were synthesized by chemical vapor deposition (CVD) with nickel and lanthanum catalysts. Characterization of the size, purity, surface area and elemental composition of the MWCNT provided by the manufacturer and an independent analysis was also performed by Millennium Research Laboratories (MRL) Inc., (Woburn, MA). Details of characterization have been previously reported by our laboratory [26,27]. Briefly, Helix reported 0.06% lanthanum by energy dispersive x-ray analysis (EDX) and MRL reported 0.03% lanthanum by inductively coupled plasma auger electron spectroscopy (ICP-AES). Using EDX, Helix detected only 0.12% Ni, yet MRL detected 5.53% Ni using the same method. However, MRL detected 0.34% Ni by ICP-AES and Helix did not detect Ni by ICP-AES. The specific surface area was determined by Brunauer-Emmett-Teller method (BET) and reported by Helix as 40 to 300 m^2^/g. This was consistent with MRL’s value of 109.29 m^2^/g. Helix described MWCNT as >95% pure, which was in agreement with >94% as determined by the contractor. Helix reported MWCNTs had an outer diamter of 10 to 30 nm that was slightly less than the MRL values of 30 to 50 nm. The 0.5 to 40 μm average length reported by Helix was consistent with the 0.3 to 50 μm range reported by MRL. The presence of LPS was determined using a *Limulus* amebocyte lysate (LAL) assay kit according to manufacturer’s specifications (Associates of Cape Cod, East Falmouth, MA). MWCNTs were sonicated in vehicle (0.1% Pluronic surfactant in PBS) for 60 min at 50 Hz in a bath sonicator (Bransonic Model B-22OOR-1, Fisher Scientific) prior to performing LAL. The maximum sensitivity of the LAL assay is 0.005 EU/ml. For all experiments with cell cultures, MWCNTs were suspended in 1% Pluronic F68 (BASF Corp., Florham Park, NJ), a biocompatible, nonionic surfactant, in PBS and dispersed in a bath sonicator for 30 min, then further diluted with PBS to achieve the desired final dosing concentration suspended in 0.1% Pluronic F68. In addition to the MWCNTs purchased from Helix, another source of MWCNTs was provided as a kind gift from from Dr. Somenath Mitra (Department of Chemistry and Environmental Science, New Jersey Institute of Technology, Newark, NJ) in either an unmodified “as-prepared” form (AP-MWCNTs) or as an acid-purified form to remove residual nickel (PD-MWNCTs). The AP-MWCNT stock was purchased as a powder from Cheap Tubes, Inc., (Brattleboro, VT). The details of acid-purification to derive the PD-MWCNT and the characteristics of AP-MWCNTs and PD-MWCNTs have been previously reported [50]. Briefly, AP-MWCNT and PD-MWCNT have an average outer diameter of 20–30 nm, a length of 10 to 30 μm, and a purity of >95%. AP-MWCNT contain 4.49% Ni, 0.76% Fe and PD-MWCNT contain 1.8% Ni, 0.08% Fe. Carbon black nanoparticles (CBNPs) were purchased from Columbian Chemicals (Marietta, GA) and had a mean diameter of 8 nm. Nickel nanoparticles (NiNPs) were purchased from Sun Innovations, Inc. (Fremont, CA) and had a mean diameter of 20 nm.

### Cell culture

The RAW264.7 macrophage cell line was purchased from ATCC (Manassas, VA, USA). Cultures of RAW264.7 cells were grown in 60 mm dishes in a humidified incubator at 37°C at 5% CO_2_ in Dulbecco’s modified Eagles Medium (DMEM) containing 10% fetal bovine serum (FBS). For experimental use the cells were grown to confluence in dishes or wells as indicated in the individual experiments. Prior to treatment with the nanoparticles the cells were rendered quiescent for 72 hr in serum-free defined medium (SFDM) comprised of Ham’s F-12 supplemented with an insulin/transferrin/selenium mixture. The duration of MWCNT or CBNP treatment was 24 hr unless otherwise indicated.

### LDH cytotoxicity assay

Cell death was measured using the LDH assay kit from BioChain Institute (Hayward, CA, catalog # K6330400) according to the manufacturer’s instructions. MWCNT or CBNP at the concentrations indicated in 200 μl SFDM were added to triplicate wells containing 2 × 10^4^ cells/well in a 96-well plate. Cytotoxicity experiments were independently replicated at least twice. Three types of controls were utilized: (1) a background control without cells and containing only medium with MWCNTs or CBNPs, (2) low control: 2 × 10^4^ cells in total 200 μl assay medium into triplicate wells, and (3) high control: 2 × 10^4^ cells/well in total 200 μl assay medium containing 1× lysis solution (provided in the kit) into triplicate cells. After 24 hrs incubation of the cells with the nanoparticles, the medium from the wells was centrifuged at 250× g for 10 min. One hundred μl of supernatant from the control wells and nanoparticle treated wells was transferred into corresponding wells of an optically clear 96-well flat bottom plate. Then 45 ul of assay mixture was added to each well and incubated at RT for 30 minutes. The absorbance of controls and treated samples was measured at 490 nm. The cytotoxicity was then determined according to the following equation: $\text{Cytotoxicity}\left(\%\right)=\left[\left(\text{sample OD minus low control OD}\right)/\left(\text{high control OD minus low control OD}\right)\right]\times 100.$

### Transmission electron microscopy

Cells in culture were treated for 24 h with MWCNTs, then scraped from dishes, pelleted, and resuspended in 4F1G (4% formaldehyde and 1% gluteraldehyde in 0.1 M PBS (pH 7.4)). Fixed cells were immobilized by adding 1:1 ratio of 4F1G and 3% agar suspension (100 μl of each). Agar immobilized cells were embedded in Spurr’s resin. Unstained thin sections were mounted on copper grids and then examined on a Philips EM208S transmission electron microscope.

### Western blot analysis

Cells were grown in 100 mm dishes and lysed in cell lysis buffer (Cell Signaling Technology, Beverly, MA) containing 1 mM phenylmethylsulfonylfluoride. Lysates were centrifuged at 12,000 *g* for 20 min, and supernatants containing 30 μg proteins were boiled in Laemmli sample loading buffer (Bio-Rad Laboratories, Inc., Hercules, CA) for 5 min and loaded on Criterion^TM^ 4-20% Tris–HCl precast gel (Bio-Rad Laboratories, Inc., Hercules, CA). After electrophoresis for 2 hr, the proteins were transferred to PVDF membranes and blocked with 5% non-fat dry milk-PBST buffer [phosphate-buffered saline (PBS) containing 0.1% Tween-20] for 1 hr at RT. The membranes were incubated overnight at 4°C with 1,000-1,500 dilution of the following antibodies: COX-2 and iNOS goat anti-rabbit polyclonal antibodies (Cayman Chemical, Ann Arbor, MI), p-ERK1/2, and ERK1/2 goat anti-rabbit polyclonal antibodies (Cell Signaling Technology, Beverly, MA). Equal lane loading was assessed using β-actin (Cell Signaling Technology). The blots were rinsed three times with PBST buffer for 10 min and incubated for 1 hr with 1:5,000 dilution of the horseradish peroxidase (HRP)-conjugated secondary antibody (Sigma-Aldrich, St Louis, MO) and then washed again with PBST buffer. The transferred proteins were visualized with an enhanced chemiluminescence detection kit (GE Healthcare UK Ltd., Buckinghamshire, England).

### PGE_2_ assay

PGE_2_ concentrations were measured using an ELISA kit (Cayman Chemicals, Ann Arbor, MI) according to the manufacturer’s instructions. Briefly, microplates coated with EIA buffer, standard, and samples were incubated overnight at 4°C. After washing with buffer, Ellman’s reagent was added to each well and the plates were maintained for 1 hr at RT in the dark. Absorbances were measured at 405 nm with an ELISA reader (Molecular Devices, Sunnyvale, CA). The amounts of PGE_2_ were calculated from the linear portion of the standard curve according to the manufacturer’s instructions.

### Nitric oxide assay

Nitric oxide production was determined spectrophotometrically using the Griess reagent kit according to the manufacturer’s instructions (Promega, Sunnyvale, CA). Briefly, 100 μl of the cell supernatant was added to each well and after the addition of 100 μl of Griess reagent to each well the absorbance at 540 nm was measured by using a microplate reader. The Griess reagent was prepared by mixing 1 part of 0.1% (w/v) naphthylenediamine dihydrochloride in distilled water plus 1 part of 1% (w/v) sulfanilamide in 2.5% H_3_PO_4_. The NO_2_^-^ concentration was calculated from a NaNO_2_ standard curve.

### Statistical analysis

All graphs were constructed and statistical analysis performed using GraphPad Prism® software v. 5.00 (GraphPad Software, Inc., San Diego, CA). A one-way ANOVA with a post-hoc Tukey test, Bonferroni test, or Dunnett’s test was used to identify significant differences among various treatment groups. Significance was set at p < 0.05 unless otherwise stated.

## Abbreviations

MWCNT: Multiwalled carbon nanotubes; CBNP: Carbon black nanoparticles; COX-2: Cyclooxygenase-2; PG: Prostaglandin; iNOS: Inducible nitric oxide synthase; NO: Nitric oxide.

## Competing interests

The author(s) declare that they have no competing interests.

## Authors’ contributions

JKL, RL, and JCB had the initial idea of performing the studies and designed the experiments with input from KSC, HCL and BSC. JKL and BSC, and KSC performed the experiments. HCL performed densitometric analysis of Western blot data. All authors read, reviewed and approved all versions of the manuscript.
